# To explore the postoperative nutritional status and factors influencing prognosis in patients with chronic constipation complicated by malnutrition

**DOI:** 10.3389/fmed.2024.1335203

**Published:** 2024-09-03

**Authors:** Le Wang, Hongliang Tian, Long Li, Chen Ye, Jiaqu Cui, Zhiliang Lin, Bo Yang, Di Zhao, Ning Li, Xiaobo Feng, Qiyi Chen

**Affiliations:** ^1^Department of Functional Intestinal Diseases, General Surgery of Shanghai Tenth People’s Hospital, Tongji University School of Medicine, Shanghai, China; ^2^Shanghai Gastrointestinal Microecology Research Center, Shanghai, China; ^3^Shanghai Institution of Gut Microbiota Research and Engineering Development, Shanghai, China; ^4^Research Institute of General Surgery, Jinling Hospital, Nanjing University School of Medicine, Nanjing, China

**Keywords:** chronic constipation, surgical treatment, malnutrition, intestinal motility, nutritional status

## Abstract

**Background:**

Many patients with constipation also suffer from varying degrees of malnutrition, and the relationship between the two conditions is a vicious cycle. Surgery is the final step in the treatment of constipation, with a success rate of up to 95%. This study aims to investigate the effects of surgical treatment on the nutritional status of patients with chronic constipation and malnutrition.

**Methods:**

A total of 60 patients with chronic constipation and various degrees of malnutrition who underwent surgery in our department from January 2020 to March 2023 were included in this study. Biochemical tests including BMI, albumin, total protein, hemoglobin, cholesterol and lymphocyte count were conducted, as well as measurements of inflammatory markers such as Interleukin-6 (IL-6), Interleukin-8 (IL-8), and C-reactive protein (CRP). Additionally, multiple nutritional risk screening scales (NRS2002, MUST, NRI, and MNA) and the prognostic nutritional index (PNI) were used to assess the nutritional status of patients before surgery, as well as at 1 month, 3 months, and 6 months post-surgery. Finally, we analyzed the factors influencing postoperative recovery outcomes in patients.

**Results:**

Compared to pre-operation, the BMI of patients significantly increased at 1 month, 3 months, and 6 months after the operation, with statistically significant differences (*p* < 0.001). Multiple nutritional risk assessment tools (NRS2002, MUST, NRI, and MNA), as well as the prognostic nutritional index (NPI), indicated a reduction in nutritional risk and improvement in nutritional status at 1, 3, and 6 months post-surgery, compared to pre-surgery levels (*p* < 0.001). The levels of albumin, total protein, and hemoglobin in patients at 1, 3, and 6 months after the surgery were significantly higher than those before the surgery (*p* < 0.001). However, there was no significant change in the number of lymphocytes. Inflammatory markers such as IL-6, IL-8, and CRP exhibited a significant decrease after the surgery, reaching normal levels at 6 months post-surgery (*p* < 0.001). Low BMI, low PNI, and low cholesterol levels are independent risk factors for patient prognosis (*p* < 0.05).

**Conclusion:**

Surgical treatment can enhance the nutritional status of constipation patients with malnutrition, which in turn promotes the restoration of intestinal motility. The patient’s nutritional status will impact the postoperative recovery outcomes.

## 1 Introduction

Chronic constipation is a complex condition with various symptoms, and its prevalence is increasing in many countries. It has become a common health issue that negatively impacts quality of life (1). Chronic constipation can be divided into primary and secondary types (2). Primary constipation is primarily caused by factors such as inadequate dietary fiber intake, sedentary lifestyle, and abnormalities in colonic propulsion and rectal emptying. On the other hand, secondary constipation is mainly a result of medication use, including drugs used for systemic diseases like hypothyroidism or Parkinson’s disease, as well as local bowel diseases like colon cancer or diverticular stenosis (3).

Intestinal motility is influenced by various factors, such as the nutritional status of the body, immune system function, nervous system activity, bile acid metabolism, intestinal mucus secretion, and the composition of the gastrointestinal microbiota. Any disruption or dysfunction in these factors can result in impaired intestinal motility (4, 5). There is a close relationship between intestinal motility and constipation. Normal intestinal motility is crucial for maintaining regular bowel function. When intestinal motility is decreased, or when there are issues such as intestinal narrowing, obstruction, intestinal nerve damage, or imbalances in neurotransmitters, it can lead to impaired intestinal motility. This results in prolonged transit time of food in the intestines, excessive water absorption, and dry and hardened stool, ultimately causing constipation. Intestinal motility refers to the periodic contraction and relaxation movements that propel food and waste through the digestive tract (6). When motility is reduced, the frequency of contractions decreases, which can lead to insufficient force to propel food and waste efficiently through the digestive tract. Additionally, it can disrupt the function of the colon, leading to increased transit time of food in the intestines. Indeed, the sympathetic and parasympathetic nervous systems play significant roles in regulating intestinal motility. Sympathetic nerves, when stimulated, can inhibit the release of neurotransmitters that are involved in promoting intestinal contractions, thus reducing the propulsive power of the intestine (7). On the other hand, parasympathetic nerves have a promoting effect on colonic motility, as they stimulate the release of neurotransmitters that enhance intestinal contractions and facilitate the movement of fecal matter through the colon (8–10). Mucus secretion plays an important role during the process of defecation, primarily involving goblet cells and mast cells distributed in the gastrointestinal tract, the goblet cells in the gastrointestinal tract produce secreted gel-forming mucins, their key feature is the ability to form a gel, protecting the intestines from infections and facilitating movement. At the same time, studies have reported that some components of traditional Chinese medicine, such as rhubarb extracts, induce mucin secretion through the aggregation and degranulation of mast cells (11). Additionally, abnormalities in intestinal ion channels can also affect the secretion, absorption, motility, and sensation of the intestines, leading to constipation, diarrhea, and irritable bowel syndrome (IBS) (12).

When patients do not respond to standard non-surgical treatments and relevant examinations indicate a clear indication for surgery, surgical treatment should be considered. In a study involving 74 patients who underwent ileorectal anastomosis after colectomy, the results were promising. The study found that 97% of these patients expressed satisfaction with the surgery, and 90% reported an improvement in their quality of life (13). However, there is limited research on the improvement of nutritional status and the impact on postoperative recovery in patients with constipation after surgery. This article aims to comprehensively investigate the improvement of nutritional status in patients with constipation and malnutrition after surgery, as well as the nutritional factors that influence postoperative recovery.

## 2 Materials and methods

### 2.1 General information

This study has been registered on Clinical Trials.gov as part of the NCT05983926 study. In this study, a total of 57 chronic constipation patients with malnutrition who had undergone surgical treatment for constipation at our hospital between January 2020 and March 2023 were included. The basic information and surgical details of the patients are shown in Table 1.

**Table 1:** Basic information of patient.

Characteristics	Overall
N	57
Gender (male/female)	13/44
Age (years)	55, 50.31 ± 15.00
Duration of disease (years)	8, 7.31 ± 1.14
Wexner constipation score (score)	18, 18.23 ± 1.21
BMI (kg/m^2^)	17.19, 17.36 ± 1.54
Type of constipation (%)	
Slow transit constipation	41 (71.93)
Outlet obstructive constipation	11 (19.30)
Mixed constipation	5 (8.77)
History of constipation-related medications (%)	
Prokinetic drugs	49 (85.96)
Psychotropic drugs	16 (28.07)
Probiotics	38 (66.67)
Traditional Chinese medicine	31 (54.39)
Surgical time (min)	149.46 ± 48.00
Postoperative hospital stay (d)	11.28 ± 3.57
Readmission rate, *N* (%)	9 (12.26%)
Patient satisfaction	53 (92.98%)
Drainage condition	
Latex tube	24 (42.11%)
Double cannula	33 (57.89%)
Postoperative pathology	
No significant abnormalities noted	25 (43.86%)
Reduction of intermuscular nerve plexus or ganglia	32 (56.14%)
Number of completely spontaneous bowel movements 1 month post-surgery (times/day)	5.12 ± 0.96
Number of completely spontaneous bowel movements 1 month post-surgery (times/day)	2.15 ± 1.14

The data for age, duration of disease, and Wexner constipation score were recorded in terms of median, mean ± standard deviation, whereas the type of constipation and history of constipation-related medications were recorded as counts (percentage).

The inclusion criteria for this study are as follows: (1) Disease duration of more than 6 years. (2) Wexner constipation score greater than 15. (3) No response to medical treatment, biofeedback, and fecal microbiota transplantation. (4) Meeting the surgical indications for subtotal colectomy. (5) All patients were informed about the study and provided signed informed consent.

The exclusion criteria for this study are as follows: (1) Patients with mental disorders or cognitive impairment. (2) Patients with malignant tumors. (3) Patients with a history of gastrointestinal surgery. (4) Patients complicated with other organ dysfunction. (5) Patients with immune system diseases.

## 3 Methods

All patients undergo nutritional assessment using various nutrition evaluation tools before and 1, 3, and 6 months after surgery. Blood samples are also taken for complete blood count (hemoglobin and lymphocyte count), inflammatory markers (CRP, IL-8, and IL-6), and other biochemical markers (albumin, total protein, and cholesterol) testing. When a patient’s bowel frequency ranges from more than three times per week to less than three times per day, and there are no other abdominal symptoms, it is assessed as cured. Conversely, when a patient’s bowel frequency is less than or equal to three times per week or greater than or equal to three times per day, or when there are abdominal symptoms such as abdominal pain or bloating, it is assessed as not cured.

### 3.1 Nutritional risk screening tools

1. Nutritional risk screening for hospitalized patients involves using the NRS-2002 assessment form, which takes into account the patients’ nutritional status, disease severity, and age. The criteria for scoring are as follows: ① Score 0: indicates a normal nutritional status. ② Score 1: suggests mild malnutrition. ③ Score 2: indicates moderate malnutrition. ④ Score 3: represents severe malnutrition.
2. The malnutrition universal screening tool (MUST) was developed by the Multidisciplinary Malnutrition Advisory Group of the British Society for Parenteral and Enteral Nutrition. It is applicable for nutritional risk screening in various medical settings. The tool aims to identify protein-calorie malnutrition and assess the risk level by considering three factors: ① BMI, ② weight loss, and ③ disease-induced reduction in food intake. Each component is assigned a score, and the total score determines the risk level: ① a score of 0 indicates low risk, ② a score of 1 indicates moderate risk, and ③ a score of 2 or higher indicates high risk.
3. The nutritional risk index (NRI) is primarily employed to assess the impact of total parenteral nutrition support in patients undergoing major abdominal and thoracic surgeries. Nutritional risk is evaluated based on serum albumin concentration and the percentage of weight loss. The formula for calculating NRI is as follows: NRI = 1.519 × albumin concentration + 41.7 × current body weight/past body weight.
4. The mini nutritional assessment (MNA) incorporates anthropometric measurements, global assessment, dietary questionnaire, and subjective assessment. Malnutrition and nutritional risk can be evaluated by assigning scores and summing them based on the specified scoring standards. The evaluation criteria are as follows: ① MNA score ≥ 24 indicates good nutritional status; ② MNA score between 17 and 24 indicates nutritional risk; ③ MNA score < 17 indicates malnutrition.
5. The prognostic nutritional index (PNI) is an index used to assess the nutritional status, predict surgical risk, and prognosis of patients undergoing surgery. Initially developed for evaluating the nutritional and immune status of patients undergoing gastrointestinal surgery, PNI assessment includes measuring serum albumin levels and lymphocyte count in the peripheral blood. The formula for calculating PNI is as follows: PNI = serum albumin (g/L) + 5 × total peripheral blood lymphocyte count (×10^9^/L).

### 3.2 Indicators of observation

The body mass index (BMI) of patients was compared before surgery and at 1, 3, and 6 months after surgery. In addition, the scores obtained from multiple nutritional risk screening assessment tools, such as NRS2002, MUST, NRI, MNA and PNI, were compared before surgery and at the same intervals post-surgery. Furthermore, the levels of albumin, total protein, hemoglobin, and lymphocyte count were compared before the operation and at 1, 3, and 6 months after the operation. The levels of CRP, interleukin-6 (IL-6), and interleukin-8 (IL-8) were also compared before the operation and at the same intervals post-surgery.

### 3.3 Statistical methods

In this study, analysis of variance (ANOVA) with least significant difference test (LSD) for homogeneity of variance and Welch test for heterogeneity of variance were used to analyze the continuous variables. The continuous variables were presented as means and standard deviations. Chi-square test was used for all categorical and ordinal variables, and they were represented using frequencies and percentages. Further, factors with statistical significance were subjected to multiple logistic regression analysis. A significance level of *p* < 0.05 was set, and SPSS version 26.0 was used as the statistical tool.

## 4 Results

### 4.1 The changes of nutritional status of patients

Compared to the preoperative BMI, the BMI of patients at 1 month, 3 months, and 6 months after the operation exhibited a significant increasing trend. NRS2002, MUST, NRI, MNA, and PNI were utilized to evaluate the nutritional risk of patients before surgery and at 1 month, 3 months, and 6 months after surgery. As shown in Table 2, the findings revealed that the nutritional risk of patients at the time of reexamination was significantly improved compared to before surgery. However, there was no significant change at the three time points of 1 month, 3 months, and 6 months following surgery.

**Table 2:** The nutritional status of patients before surgery and at 1, 3, and 6 months after surgery, including BMI and different nutritional risk screening assessment tools (NRS2002, MUST, NRI, MNA, and PNI).

	Pre-operation	1 month after operation	3 months after operation	6 months after operation	p
BMI (kg/m^2^)	17.36 ± 1.54	19.02 ± 4.15^*^	18.68 ± 3.22	20.33 ± 3.46^****^	0.003
NRS2002	2.72 ± 0.61	0.93 ± 1.19^****^	0.75 ± 0.89^****^	0.75 ± 0.88^****^	<0.001
MUST	2.44 ± 0.9	1.14 ± 1.01^****^	1.125 ± 0.99^***^	0.67 ± 0.89^****^	<0.001
NRI	92.99 ± 10.5	109.15 ± 12.8^****^	106.42 ± 7.42^*^	109.95 ± 21.36^****^	<0.001
MNA	16.20 ± 1.42	19.46 ± 1.83^****^	20.63 ± 3.50^****^	21.57 ± 2.45^****,#^	<0.001
PNI	42.61 ± 6.75	45.68 ± 13.75^**^	47.98 ± 4.76^*^	50.28 ± 5.42^****^	<0.001

*Compared with preoperative, *p* < 0.05, **compared with preoperative, *p* < 0.01, ***compared with preoperative, *p* < 0.001, ****compared with preoperative, *p* < 0.0001, and #compared with 1 month before surgery, *p* < 0.05.

### 4.2 Changes in biochemical test results

Biochemical tests were conducted before surgery and at 1, 3, and 6 months after surgery. As shown in Table 3, the findings indicated that compared to the preoperative levels, the levels of albumin, total protein, and hemoglobin exhibited a significant increasing trend (*p* < 0.001). On the other hand, the levels of CRP, IL-6, and IL-8 showed a decreasing trend, which was statistically significant (*p* < 0.001). However, there was no significant change observed in the lymphocyte count before and after surgery.

**Table 3:** The biochemical results of the patients before surgery and at 1, 3, and 6 months after surgery.

	Pre-operation	1 month after operation	3 months after operation	6 months after operation	p
Albumin (g/L)	36.01 ± 6.72	41.63 ± 5.57^***^	41.88 ± 3.57^**^	42.94 ± 4.04^****^	<0.001
Total protein (g/L)	62.52 ± 7.71	78.41 ± 6.20^****^	78.96 ± 5.97^****^	74.51 ± 7.07^****^	<0.001
Hemoglobin (g/L)	110.26 ± 13.99	120.21 ± 13.57^*^	132.88 ± 13.91^****,#^	125.75 ± 9.09^***^	<0.001
CRP (mg/L)	16.20 ± 1.42	2.32 ± 1.40^****^	3.64 ± 2.19^**^	1.76 ± 1.30^****^	<0.001
IL-6 (pg/ml)	23.29 ± 28.49	5.14 ± 4.95^**^	4.76 ± 4.21^*^	2.36 ± 0.96^****^	0.009
IL-8 (pg/ml)	80.11 ± 71.59	9.16 ± 15.09^****^	7.44 ± 9.03^***^	6.10 ± 7.17^****^	0.006
Lymphocyte (*10^9^/L)	1.34 ± 0.58	1.52 ± 0.57	1.22 ± 0.35	1.53 ± 0.54	0.342

*Compared with preoperative, *p* < 0.05, **compared with preoperative, *p* < 0.01, ***compared with preoperative, *p* < 0.001, ****compared with preoperative, *p* < 0.0001, and # compared with 1 month after surgery, *p* < 0.05.

### 4.3 Relationship between nutritional status and surgical outcome

An analysis of factors affecting clinical recovery was conducted, and it was found in Table 4 that preoperative BMI, NRI, cholesterol levels, and lymphocyte count may influence the prognosis. Multivariable logistic regression analysis revealed that low BMI, low PNI, and low cholesterol levels are independent risk factors for patient prognosis (*p* < 0.05) (Table 5).

**Table 4:** Factors influencing postoperative recovery in univariate analysis.

Independent variable	Group	Dependent variable	
	(*N* = 57)	Clinical cure rate	p
BMI (kg/m^2^)	≤17.5	73.33% (22/30)^*^	0.0271
	>17.5	96.30% (26/27)	
NRS2002	≤2	89.66 (26/29)	0.2973
	>2	78.57% (22/28)	
MUST	≤2	88.89% (24/27)	0.4761
	>2	80% (24/30)	
PNI	≤44	74.07% (20/27)	0.07
	>44	93.33% (28/30)	
NRI	≤94	70.97% (22/31)^**^	0.0026
	>94	100% (26/26)	
MNA	≤16	75.86% (22/29)	0.1443
	>16	92.86 (26/28)	
Cholesterol (mmol/L)	≤4.5	71.43% (20/28)^*^	0.0119
	>4.5	96.55% (28/29)	
Total protein (g/L)	≤63	79.31% (23/29)	0.4703
	>63	89.29% (25/28)	
Albumin (g/L)	≤35	76.67% (23/30)	0.1494
	>35	92.59% (25/27)	
Lymphocyte (*10^9^/L)	≤1.1	72.41% (21/29)^*^	0.0253
	>1.1	96.43% (27/28)	
Hemoglobin (g/L)	≤115	79.31 (23/29)	0.4703
	>115	89.29% (25/28)	
CRP (mg/L)	≤10	93.33% (28/30)	0.07
	>10	74.07% (20/27)	

*p < 0.05 and **p < 0.01.

**Table 5:** Factors influencing postoperative recovery in multiple logistic regression analysis.

Variable	Multifactor analysis		
	OR	95%CI	p
BMI	35.982	4.582–282.521	0.001
NRI	40.900	5.315–314.727	<0.001
Cholesterol (mmol/L)	9.572	1.030–88.947	0.047
Lymphocyte (*10^9^/L)	7.527	0.950–59.628	0.056

## 5 Discussion

Patients with chronic constipation often experience symptoms such as abdominal pain, abdominal distension, and reduced digestion and absorption function. This leads to an inadequate fulfillment of the body’s nutritional requirements, resulting in a worsening of the patient’s nutritional status (14). Concurrently, long-term malnutrition in patients can cause atrophy and degeneration of the gastrointestinal mucosa, reduction in the number of nerve cells in the muscular layer, decreased surface area of the small intestine, and impaired intestinal motility function. These factors contribute to the exacerbation of constipation. The relationship between the nutritional status of the patient and intestinal motility is closely interlinked, with each factor influencing the other (15). Hence, it is crucial for patients aiming to restore intestinal function to maintain good nutritional status. Based on our data, it can be observed that low BMI, low NRI, and low cholesterol levels can affect the prognosis of surgery. This also reflects a certain relationship between the recovery of intestinal motility and the nutritional status of patients.

Low BMI, low NRI, and low cholesterol may impact postoperative prognosis for the following reasons. Low BMI may indicate lower muscle mass in patients. Intestinal motility relies primarily on the smooth muscle movements of the intestinal wall. The reduction in muscle mass can potentially affect the motility of the intestines, resulting in delayed recovery after intestinal surgery (16). NRI is positively correlated with body weight gain and the amount of body protein. Protein, as an important carrier, is responsible for transporting nutrients and oxygen to various parts of the body, including the intestines. Low levels of protein may lead to insufficient supply of nutrients and oxygen, affecting the regeneration and repair of intestinal cells, thereby influencing the recovery of postoperative intestinal motility. Additionally, low protein levels may be associated with fluid imbalance, leading to intestinal mucosal edema and reduced motility, further affecting the recovery of postoperative intestinal motility (17). Low cholesterol levels can potentially result in inadequate synthesis of bile acids, which in turn affects the digestion and absorption of fats, thus impacting the recovery of postoperative intestinal motility (18). Low cholesterol levels may also lead to unstable cell membranes and impaired cellular function, potentially affecting normal intestinal cell function and disrupting intestinal motility recovery. Furthermore, low cholesterol levels may result in insufficient hormone synthesis, which can affect the normal function of the intestines and the digestive system.

The gastrointestinal tract serves as not only a crucial organ for digestion and absorption but also the largest immune organ in the body. It houses approximately 60–80% of the body’s general immune cells, which play a vital role in maintaining intestinal immune homeostasis (19, 20). Constipation causes the colon to experience chronic obstruction, leading to prolonged transit time of feces through the intestine. This, in turn, results in an increased concentration of various bacteria in the fecal matter (21, 22). Prolonged contact between the colonic mucosa and fecal matter can promote the growth and multiplication of opportunistic or pathogenic bacteria in the intestine. This disrupts the balance of intestinal flora and displaces a significant number of bacteria (22). Additionally, it can lead to increased protein catabolism and reduced turnover of the colonic mucosa, compromising the integrity of the intestinal barrier (19). Consequently, this triggers the activation of inflammatory factors such as CRP, IL-6, and IL-8, while also suppressing the body’s immune response (23). The results of this study indicate that there was no significant change in lymphocyte count post-surgery compared to pre-surgery. Lymphocytes are a complex and heterogeneous group of cells that originate from multipotent hematopoietic stem cells in the bone marrow, which then differentiate into lymphoid stem cells, proliferate, differentiate, and mature in various parts of the body. These cells generally exhibit significant changes in infectious diseases, and in the context of surgical treatment, noticeable changes typically occur only after tissue transplantation due to rejection reactions (24, 25).

After undergoing surgical treatment, the patient’s constipation was effectively resolved, leading to the restoration of intestinal motility. Consequently, the patient’s nutritional status showed gradual improvement, thereby breaking the detrimental cycle between constipation and malnutrition. Thus, surgical treatment plays a crucial role in addressing constipation in patients with malnutrition. Our data indicates that the nutritional status of patients significantly improved during the 1 month, 3 months, and 6 months follow-up periods after the surgery, as compared to pre-surgery levels. We observed statistically significant changes in various protein levels (albumin, total protein, and hemoglobin), inflammation-related markers (CRP, IL-6, and IL-8), nutritional risk screening scores (NRS2002, MUST, NRI, and MNA), and prognostic nutritional index. However, there was no significant trend in the changes observed at 1 month, 3 months, and 6 months after surgery, suggesting that the main improvement in nutritional status occurred within the first month post-surgery. Possible reasons are as follows: within a month after surgery, the intestinal tissues of the surgical incision gradually heal. The newly formed tissues and the structure of the anastomosis are stable, which is conducive to the restoration of normal intestinal movement and peristaltic function (26, 27). The damaged nerves gradually recover, and the function of the intestinal neural network also recovers, thereby promoting the restoration of intestinal motility (28). At the same time, the inflammatory response gradually decreases, and the concentration of inflammatory mediators decreases (29). This helps to reduce the level of intestinal inflammation, improve intestinal motility, promote gastrointestinal digestion and absorption function, and improve the patient’s nutritional status.

## Data Availability

The original contributions presented in the study are included in the article/supplementary material, further inquiries can be directed to the corresponding authors.
